# THE IMPACT OF HIIT ON MUSCULAR, ENDURANCE, AND QUALITY OF LIFE METRICS IN OLDER ADULTS

**DOI:** 10.1093/geroni/igae098.4294

**Published:** 2024-12-31

**Authors:** Rashelle Hoffman, Blake Murphy, April Krywe, Devon Stoffel, Mitchel Magrini

**Affiliations:** Creighton Univeristy, Creighton University, Nebraska, United States; Creighton University, Omaha, Nebraska, United States; Creighton University, Omaha, Nebraska, United States; Creighton University, Omaha, Nebraska, United States; Creighton University, Omaha, Nebraska, United States

## Abstract

High-intensity interval training (HIIT) can mitigate age-related declines in muscle mass and strength that lead to decreased functional ability and loss of independence. However, previous HIIT protocols are often treadmill or recumbent bike-based with limited whole-body functional exercises and often do not include older adults (OA) beyond 70 years of age. The aim of this study was to examine the effects of a 12-week, Total Body HIIT (TB-HIIT) protocol on muscle size (cross-sectional area [CSA] of the rectus femoris [RF], vastus lateralis [VL], and vastus medialis [VM]), muscle strength (peak toque [pT] and rate of torque development [pRTD]), muscle activation (peak electromyography [pEMG] and rate of EMG rise [pRER]), cardiovascular endurance (6-minute walk test [6MWT]), and quality of life (QOL, 36-Item Short Form [SF-36]) in OA. Older adults, 65-95 years of age, were recruited and completed all outcomes at baseline and following the 12-weeks of TB-HIIT. The TB-HIIT protocol progressively increased the exercise-to-rest ratio every four weeks (20sec:40sec, 30sec:30sec, 40sec:20sec) with target intensity being 85-95% of participants’ heart rate max. Twenty-four OA (74.8□6.37 years, range=65-89) completed this study. Results showed significant increases in VL-CSA (p=0.02, g=-0.77), VM-CSA (p< 0.001, g=-1.03), cardiovascular endurance (p< 0.001, g=-0.95), and health-related QOL (p=0.012, g=-0.47). No significance was revealed in RF-CSA (p=0.068, g=-0.4), pT (p=0.78, g=-0.17), pRTD: p=0.91, g=-0.07), pEMG (p=0.77, g=-0.17) or pRER (p=0.51, g=-0.39). Future research should continue to explore the benefits of TB-HIIT with consideration towards OA subpopulations with comorbidities and greater physical impairment to advance exercise prescription and function for this population.

